# Older Adults’ Experiences Navigating Setup of Digital Health Technology: Implementation Report

**DOI:** 10.2196/70319

**Published:** 2026-05-04

**Authors:** P Jeffrey Brady, Rachel McCloud, Erin Higgins, Aishwarya Mahesh, Keith LeJeune, Jon Black, Anil Singh

**Affiliations:** 1 Highmark Health Pittsburgh, PA United States; 2 The Doctors Company Napa, CA United States; 3 John Snow, Inc Boston, MA United States; 4 Allegheny Health Network Pittsburgh, PA United States

**Keywords:** digital health technology, older adults, user-centric design, ethnographic interviews, telehealth

## Abstract

**Background:**

Digital health and connected technologies may support better health outcomes among older adults, including those with multiple chronic conditions or low engagement in health behaviors. However, initial experiences with technology, including during unboxing, setup, and first use, can influence emotional reactions and perceptions and can ultimately determine sustained, meaningful use. Older adults with low technology experience or poor health may be particularly vulnerable to frustration, stress, or abandonment of devices when early interactions are negative.

**Objective:**

The purpose of this implementation study was to closely observe the initial engagements with a telehealth treatment app and connected blood pressure monitor (BPM) among a group of older adults with low prior technology use and reported low health behavior engagement. The goal was to identify setup “pain points” that may influence initial impressions and intention to use the technology over time.

**Methods:**

A total of 24 older adults (aged ≥65 years) were recruited for a 4-week trial of a telehealth app. Participants were provided with a box containing a tablet preloaded with the app, paper instructions, and a BPM and cuff. Researchers first conducted in-home ethnographic interviews with participants to observe the unboxing and setup process, documenting experiences with reading instructions, using the BPM, and engaging with customer support. Weekly check-in calls and a final exit interview captured ongoing experiences and likelihood of continued use. Interview recordings were transcribed and independently coded, guided by the unified theory of acceptance and use of technology.

**Results:**

Most of the sample were White (20/24, 83%) and female (14/24, 58%). Negative experiences with the app’s customer support were the top challenge for participants, with representatives providing confusing steps or conflicting terminology. Other common challenges were understanding instructions, connecting to Bluetooth, and correctly using the BPM. While 67% (16/24) of the participants indicated that they were likely or very likely to continue to use the app after the study ended at the end of week 1, this number dropped to 54% (13/24) by the end of the 4 weeks. Participants who reported lower technology self-efficacy at the beginning of the study also experienced frustration, anxiety, and embarrassment as friction with the setup process continued.

**Conclusions:**

First impressions of digital health apps play a critical role in influencing older adults’ emotions and perceptions regarding the technology and may impact the likelihood of longer-term engagement. Those with lower technology self-efficacy are particularly susceptible to experiencing negative emotions such as frustration, stress, or shame. Mobile health apps and interventions targeting older adults should incorporate simplified instructions with clear, consistent terminology and well-trained customer support staff to improve the onboarding experience.

## Introduction

New advances in digital technology show great promise in providing safe and convenient alternatives to in-person health care and promoting enhanced monitoring capabilities for older adults [1,2]. This remote monitoring may include the ability to facilitate aging in place and early detection of health status changes [3]. Despite concerns about historically low adoption of mobile electronic devices to support medicine and public health, or mobile health (mHealth), by older adults [4], engagement has increased markedly over time [1], especially when these technologies are perceived as easy to use and beneficial for health [5].

Older adults have different use patterns and preferences for mHealth compared to their younger counterparts [6]. There remains a need for patient-centered designs and solutions to maximize adoption and positive user experience among older adult populations [1]. Remote health technologies that are too complex may exacerbate the treatment burden of older adults, particularly for those with lower technology self-efficacy [7]. There are also differences along socioeconomic lines, with low-income populations exhibiting lower mHealth adoption [8].

Recent reviews identify factors that must be considered when designing health apps and connected devices. For example, Wildenbos [9] names 4 main age-related barrier categories that increase the challenge of interacting with technological devices: cognitive ability, physical ability (eg, dexterity), visual or audial impairments, and motivation [9]. Wang et al [3] elaborate on these categories, identifying factors that contribute to the ideation, design, and evaluation of health apps, ranging from patient characteristics and perceptions to technology flow and features. The authors propose the conceptualization of the digital patient experience as a unique sum of experiences, including how interactions are framed by both technological characteristics and a patient’s background and behavioral determinants.

There may be a range of reactions to and perceptions of mHealth by older adults that span beyond technical attributes and can intersect with health-related knowledge, behaviors, and beliefs [10]. mHealth technologies require a range of skills to use, which may be challenging for those with complex care needs that may place more demands on their mental capacity [11]. Older users are compelled to undergo an ongoing process of cognitive and social adaptation to technology as technologies upgrade and evolve [12]. Older adults may also be subject to a stereotype threat described by Mariano et al [13], whereby they may underuse computer technology due to their fear of confirming negative stereotypes of their social group, which may lead individuals to distance themselves from the stereotyped domain. Accessing and learning how to use a new smartphone app or learning how to operate a related medical device may involve enhanced cognitive work to integrate the solutions into their daily lives, which can lead to frustration or stress [7] and, ultimately, abandonment of the technology.

Specific types of mHealth technologies and features may present distinct challenges for older people. For example, prior research has demonstrated that older adults have also reported generalized feelings of technological anxiety [2], with complexity concerns having emerged as a main stressor [12]. Furthermore, prior research has reported risks associated with health information tracking, which may trigger negative emotions among patients with multiple chronic conditions and potential emotional draining in this group [14].

Furthermore, the focus on health-related apps introduces another level of consideration as an individual’s health-related perceptions, beliefs, and behaviors may intersect with their technology views to influence uptake. For example, Bertolazzi et al [15] illustrate the range of health-related barriers that have been identified within health technology studies, including cognitive decline, fear of being judged by their physicians for their health behaviors, and hesitance of being reminded of the negative aspects of their illness. Furthermore, older adults with poor health, such as complex conditions or comorbidities, have a lower likelihood of adopting health technology. Older adults may also feel overloaded by information about their condition, leading to increased anxiety and more resistance to the technology [16], with mental health conditions such as depression and anxiety impacting both chronic disease self-management [17] and technology engagement [16].

Although these reactions have been cited across studies, many lack the granularity to identify the specific components—or accumulation of components—that lead to frustration or stress. Given the range of potential emotions that can influence technology adoption among older adults, it is important to understand the pain points in initial engagement that are evident when observing engagement in real time during these initial stages. Given that many older adults may already have health challenges and/or barriers to healthy behaviors at the time of first adoption, it is critical to determine whether this group is receptive to mHealth and able to engage with its content.

The purpose of this implementation report is to explore in depth the reactions and barriers to initial engagement with a telehealth and blood pressure monitoring app among a group of older adults who had low previous technology engagement and poor health behaviors. This study aligns with the “implementation for persons” classification of digital health studies by the World Health Organization [18], specifically piloting and generating evidence regarding aspects of personal health tracking. The researchers aimed to observe participants engage with each individual phase of the app introduction to understand where challenges arise to serve as the first stages of a larger program of research involving the rollout of health apps to this population. Detailing each part of this process may illuminate key frustration points that may be addressed in future app design and rollout.

## Methods

### Overview

This qualitative implementation project was led by a large health care organization with operations that include health insurance, health care delivery, population health improvement activities, and the provision of mHealth services. A central aim of this study was to determine the feasibility of introducing apps that have options for virtual visits and vital sign monitoring for priority populations (older adults with mental health conditions and low health management). The main feature of the implemented app was the ability to provide telehealth visits and a physician’s advice. The blood pressure monitor (BPM) and cuff were added to understand the feasibility of asking older adults to participate in remote monitoring activities.

This 4-week study used an approach similar to that of past studies with older adults and technology use [19,20]. Researchers first conducted an ethnographic interview, a form of field research that relies on direct observation in the participants’ home environment, as well as field notes and interviewing. Observations captured the initial reactions of older adults as they began to engage with an app and connected BPM and cuff situated within the context of their home environment. If participants had difficulty with setup, they were first asked to call the app company’s technical support number to ask for assistance. After noting any encounters with technical assistance, the study staff members assisted the participants with setup if they were still unable to operate the app. Brief weekly check-in phone calls took place at the beginning of weeks 2 to 4 to understand any challenges to app use during that period, and a final 60-minute phone interview gathered information about participants’ overall perceptions and experiences using the app.

### Sample and Recruitment

Recruitment and data collection were performed by a market research firm located in Pittsburgh, Pennsylvania, that has researchers experienced in conducting qualitative and ethnographic interviews with an emphasis on context. The firm maintains a list of potential participants for their market research survey panel. Members of the market research firm first invited eligible research panel members via email and phone. The firm also conducted outreach efforts, including flyers, cold calls, word-of-mouth recommendations, and a social media advertisement. All recruitment efforts were undertaken in nonclinical, community settings. Potential participants were sent an email invitation to be officially screened for the study. Screening questionnaires were administered over the phone or via email to determine eligibility for the study. Recruitment efforts continued until at least 20 to 25 participants were enrolled (final participant number: 24).

This was an adjunct study of a larger project on technology and older adults, which dictated the scope of the inclusion criteria. These criteria included individuals who were aged ≥65 years, residing in southwestern Pennsylvania, and members of a Medicare Advantage plan and self-identified as having at least one of the following conditions: depression, anxiety, bipolar disorder, obsessive-compulsive disorder, posttraumatic stress disorder, or substance use disorder. Eligible participants were also required to indicate a low level of engagement with their health and technology use, including not taking steps to improve their health and wellness, and infrequent to no use of health-related technologies. Finally, individuals had to agree to an initial in-home ethnographic interview and a 4-week trial of the app and BPM.

### Ethical Considerations

The Allegheny Health Network Institutional Review Board (2024-262) approved this research study. The study interviewer introduced the consent form to the participants at the beginning of the in-home ethnographic interview. The interviewer walked through the consent form information with participants and then had them sign before continuing. Transcripts of these interviews and subsequent surveys were deidentified prior to analysis. Participants were compensated with US $100 for each week they completed a survey about their experience (US $400 in total). All videos, related transcripts, and feedback notes were stored via a secure server on a password-protected computer. While participants did not receive transcripts of their responses, they were able to access all information entered through the app (eg, blood pressure readings, in-app messages, or activities). These health-related responses were kept secure and were not shared with the research team. The data gathered from this study and related intellectual property are owned by the health care organization.

### Provision of Materials

Participants were given a package that included a study introduction letter, a one-page instruction sheet from the app manufacturer, and a box with a tablet preloaded with an app that offered home-based telehealth options and a BPM and cuff. Participants were instructed to (1) open the box, (2) examine the contents (including technology and any instructions), (3) sign into the app, (4) engage with the content, and (5) take an initial blood pressure reading.

### Data Collection

#### Initial Ethnographic Interview

During the introduction to the app and related technology, a research staff member sat with the participants and documented the process stated above, asking participants to think aloud as they completed each activity. They also observed any encounters with customer support, observing the call and then asking the participants about their reaction to the help they received.

At the time of unboxing, researchers conducted a 120-minute in-home interview focused on initial impressions of the app and any potential challenges faced. They observed how participants unboxed and engaged with the technology and its instructional components. Participants were asked to think aloud throughout the process, voicing any reactions (both positive and negative) and questions about the technology. In addition, researchers documented the environmental factors (eg, state of the home, including clutter and medication storage); the location in the home selected for tablet use (eg, at the kitchen table or in the living room); and any other contextual factors present, such as others living in the home. Semistructured interview guides and topics for ethnographic observation for this process followed an adaptation from the unified theory of acceptance and use of technology (UTAUT) [21]. Interview questions focused on 3 of the 4 domains of the UTAUT: effort expectancy (how difficult or easy the app would be), performance expectancy (if they believed the app would bring joy to their everyday life), and facilitating conditions (what in their environment would be a barrier to or facilitator of app use). An additional measure asked about future likelihood (intention) of using the app beyond the initial study period, with answer choices ranging from “not at all likely” to “very likely.” Questions were also guided by findings from initial formative interviews with participants that revealed previous health and technology barriers (to be published elsewhere).

#### Weekly Check-Ins

Over a 4-week period, researchers conducted a series of brief weekly check-in phone calls with participants to ask about ongoing perceptions of app utility or challenges experienced when using the app. Participants were also asked whether they had contacted customer support for help during that week and what their experiences had been with this interaction.

#### Exit Interview

At the end of week 4, researchers conducted a 60-minute exit interview over the phone to assess overall perceptions of the app and participants’ use experience and repeated the question of likelihood of continuing to use the app.

### Analysis

Recordings from each phase of the research process (initial ethnographic interviews, phone check-ins, and exit interview) were transcribed. Codebook development was iterative, with initial codes based on UTAUT domains and then expanding as new themes emerged [22]. Two trained qualitative analysts independently coded the transcripts, coding each phase separately. Codes within a particular phase were first grouped into themes that aligned with UTAUT domains and the proposed extensions found in our formative research. We then used inductive coding to allow for the identification of any emergent themes, particularly those related to health-related topics. As this process continued, themes regarding emotional reactions (eg, feelings of uncertainty, anxiety, and happiness) emerged as another topic to include in the coding scheme; the analysts then analyzed all the transcripts to identify all content related to emotions. The coding process continued until an agreement was reached between coders. The coders repeated a similar process on all observations and field notes, emphasizing the facilitating conditions construct of the UTAUT.

## Results

### Overview

Most of the sample was female (14/24, 58%) and White (20/24, 83%) and had a household income of less than US $60,000 (Table 1). Participants reported a range of age, all older than 65 years, and predominantly self-reported depression or anxiety.

**Table 1:** Participant demographics and other characteristics from a mobile health feasibility study with older adults in Pennsylvania (n=24).

Variables			Participants, n (%)
Female			14 (58)
**Age group** **(y)**			
	65-67	5 (21)	
	68-70	8 (33)	
	71-75	5 (21)	
	≥76	6 (25)	
**Race**			
	Asian	1 (4)	
	Black	3 (13)	
	White	20 (83)	
**Household income (US $)**			
	<60,000	15 (63)	
	60,000-99,000	5 (21)	
	100,000-249,000	2 (8)	
	Preferred not to answer	2 (8)	
**Reported mental health conditions^a^**			
	Depression	15 (63)	
	Anxiety	10 (42)	
	PTSD^b^	1 (4)	
	OCD^c^	1 (4)	
	Substance use disorder	1 (4)	

^a^Some reported more than one condition.

^b^PTSD: posttraumatic stress disorder.

^c^OCD: obsessive-compulsive disorder.

### Challenges Observed With Technology Setup

Several challenges were observed throughout the unboxing and setup process (Table 2), with device setup notably more difficult for those with little or no previous experience with digital technology. Many older adults expressed anxiety that they might make a mistake that would break the tablet. Participants experienced difficulty with pairing the BPM with the app, including specific issues related to awareness and understanding of Bluetooth functionality, finding the Bluetooth icon, and/or not knowing how long to press the button to activate it.

**Table 2:** Observed challenges during the unboxing and setup process.

Issue			Suggested fix
**Unboxing**			
	Older adults may put aside the instructions.	Put all instructions together and have them clearly labeled.	
**Opening the device**			
	Frustrations with difficult packaging	Ensure that packages are easy to open for those with limited dexterity and hand strength.	
**Reading instructions**			
	Small font was difficult to read.	Use a large font with simple icons.	
	Inconsistent language throughout the document led to confusion and frustration.	Be consistent with the language used in instructions and on the devices. Provide one set of comprehensive, step-by-step paper (not video) instructions that cover all required steps and devices (including Wi-Fi and battery setup) from unboxing to the actual stage of use. Explain both “what to do” and “how to do it” in the instructions. Any action required needs written step-by-step and very literal directions.	
**Device setup**			
	Older adults did not have their Wi-Fi password nearby, which led to frustration and paused the step.	Ensuring that older adults have their Wi-Fi password and know how to set up a device on Wi-Fi; this should be incorporated as a first step and explained in detail.	
	Identifying the Bluetooth button on the BPM^a^ was difficult for some. Knowing how long to press and hold also created errors and frustrations.	Provide clear, step-by-step instructions for both what to do (eg, “pair the device”) and how to do it (eg, “look for an icon that looks like X and press it for 5 seconds”).	
	Lack of consistency in the terminology used in the instructions, on the device screen, and by customer support representatives made setup very confusing.	Use terminology that is consistent across all modalities to ensure comprehension (eg, “on” and “off” vs “start” and “stop” were used interchangeably).	
	Pop-up messages requesting permission to access contacts during video call setup caused feelings of doubt and anxiety regarding security.	Limit the functionality that requires the device to access the user’s personal information. If this is necessary, communicate the purpose and benefits clearly and in plain language.	
	On-screen instructions would give multiple steps at the same time, creating confusion on the order and actions involved.	Communicate the required actions one at a time, not all at once.	
**BPM use**			
	Many participants misused the device on their first attempts. Most were unable to place the BPM cuff on their arm correctly.	Reinforce correct actions with messages indicating progress to reduce anxiety and encourage engagement.	
	One-third of the older adults did not know what a normal blood pressure should be, even if theirs was out of range.	Supporting materials and information on when to contact a physician need to be provided.	

^a^BPM: blood pressure monitor.

Improperly placing the BPM cuff was also a frequently observed error, although this placement was not confirmed during customer support calls. Furthermore, over a third of the participants were unable to interpret their blood pressure reading, which heightened stress and diminished confidence.

Several participants were observed putting aside or misplacing paper instructions during the initial unboxing and then exhibiting frustration when they did not know how to proceed. Inconsistent language across paper instructions and on-screen instructions also caused confusion, exacerbated by different descriptions used by customer support staff when troubleshooting with participants. Directions often said what the participant should do, such as pressing the Bluetooth button, but did not give the level of direction needed (such as a description of what the button looked like) for them to fully execute the request. Other vision and dexterity challenges, such as small font and difficult-to-open packaging, were noted for nonapp components.

### Customer Service

When initiating a first video call through the app, participants frequently noted their confusion with which buttons to press and in which order. Customer service calls often involved wait times of 10 minutes or more but with little indication that the participant was still on hold. One participant expressed the desire for hold music or some sign that the call was still connected, whereas another suggested a periodic message acknowledging the delay.

Engagement with customer service was difficult for most participants. Inability to clearly hear the customer service representative was a common challenge. Additionally, study staff observed that customer service representatives often did not ask questions in an approachable way that participants could fully understand. Research staff noted the lack of consistency in the terminology that representatives used, which often differed from the terms used in the instructions provided. For example, most representatives did not ensure that the BPM was “paired with the app,” instead asking whether the devices “were connected.” This slight difference led to confusion and participants answering “yes” when they did not understand the meaning of the question. One participant said that this was to avoid feeling “dumb.” It was observed across interactions that customer service representatives did not check to determine whether the blood pressure cuff was put on correctly. In another instance, the customer service representative asked the participant whether there was someone else they could speak with, causing the participant to feel incompetent and frustrated.

### Emotional Impact of Technology Use

**Figure 1:**
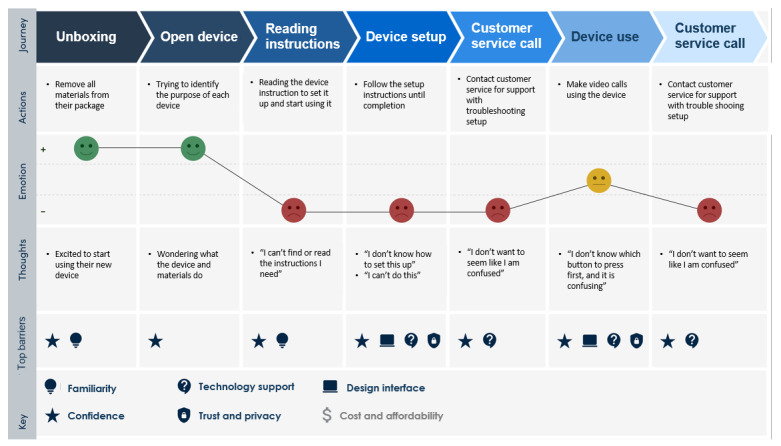
Graph of the emotional states most often experienced by participants through their initial app and blood pressure monitor use.

Older adults who had more experience with technical devices were more confident and completed setup faster than those who were less technologically savvy; setup led to anxiety for many older adults without technology experience. Although many expressed excitement during the initial unboxing, more negative reactions arose as the setup process became confusing, with continued friction causing anxiety to build (Figure 1). Participants named a range of emotions as challenges impacted setup and use, including shame, stress, and anxiety (Textbox 1).

**Participant quotes illustrating the range of emotions identified.**

**Shame**

- “I will be judged by the universe as being pitifully stupid, shameful, can’t figure it out. Then it reminds me of all the other things that I haven’t been able to figure out that I try to ignore and leave behind. There’s not a lot of confidence in my own efficacy around this kind of stuff.”
- “I just feel a little inept right now. I should be able to figure this out, but why I’m having so much trouble getting it started. I don’t know what I’m looking for. I think they could improve their system, but I have to take responsibility in the fact that I should be catching on quicker than I am on this.”

**Frustration and anger**

- “When you purchase something, you want to start using it. And I couldn’t do that with the information that’s here. I’m frustrated because there’s no true instructions on how to use it. And you get frustrated and angry. You put out your money to buy something, and you can’t use it. And then you can’t even talk to a person on the phone to walk you through it and tell you what to look for. So, you have to wait for an e-mail. And are you going to understand what comes back from the e-mail? Because it’s going to be words too.”

**Fear or anxiety**

- “My daughter says, if you don’t know what to do, just try something and see what happens. I’m afraid of trying anything because I might completely screw the thing up. I’m not going to randomly push the blue arrow or whatever unless I’m told to do that.”
- “It needs to be absolutely crystal clear user-friendly as possible. Because when you are not feeling well, your anxiety level goes up.”

**Stress**

- “My blood pressure is typically not that. I don’t think that’s indicative of this whole setting. Any physician worth his salt would be ‘It’s high blood pressure, Mark.’ So, when you’re not in the office with a physician, you’re in an unsafe place. And I think that might play on the psyche, which leads to higher blood pressure. That’s crazy 155 over 105. I’ll call my doctor, but I’ll wait a little, maybe an hour or so, and give it a chance to calm down, because I’ve been setting this up, and I don’t have the best temperament.”

### Overall App Impressions

**Figure 2:**
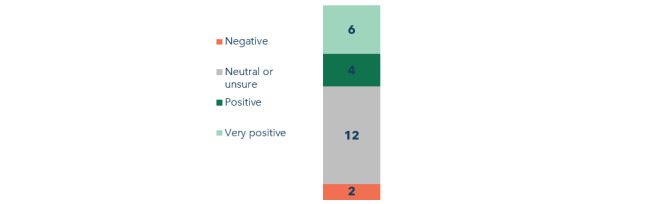
Initial impressions of the app after 1 week of use.

**Figure 3:**
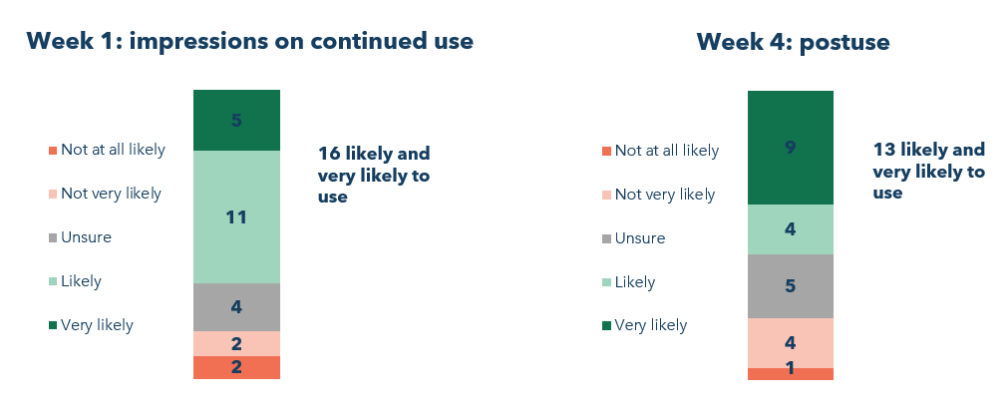
Likelihood of using the health app for care after the study ended: week 1 vs week 4 response.

After the initial experience setting up the app, participants had mixed reactions when responding to the question about their overall experience. In total, 8% (2/24) of the participants rated it as a negative experience, whereas half (12/24, 50%) were unsure (Figure 2). The remainder (10/24, 42%) had a positive or very positive initial experience with the app. When asked how likely they would be to use the app for health care at the end of week 1, a total of 67% (16/24) of the participants replied that they were “likely” or “very likely” to continue using the app after the end of the study (Figure 3). By the end of the study, this number had decreased to 54% (13/24).

## Discussion

### Principal Findings

This study examined the initial engagements and reactions of 24 adults aged ≥65 years with a tablet preloaded with a health visit app and link to a BPM. Study staff held 120-minute in-home ethnographic interviews with participants to gather their initial impressions when unboxing and beginning to use the app and blood pressure monitoring equipment. Overall, several difficulties were observed over the course of the unboxing and device setup, with particular device challenges of following specific setup instructions (eg, pairing the device) and using the BPM and cuff correctly. Setup challenges were exacerbated by lack of clarity from both paper instructions and customer service representatives’ helpfulness and empathy. Although participants reported excitement at the beginning of unboxing, those with less technology experience began to feel anxiety as setup began. Other common emotions were shame, frustration, and a specific fear of damaging or breaking something within the setup process.

This study illustrates that, even though many broader technology-based barriers are documented in the literature that deals with pre– and post–technology adoption time points, there is value in gathering data from every step of initial unboxing and setup to identify specific challenges during this time frame. Some older adults in this study experienced mounting frustration and anxiety as the setup process continued, and they were not able to understand directions or complete specific setup actions. Fear of breaking the device has been previously documented in the literature as a barrier for older adults [23], which can lead them to hesitate to experiment with or explore new apps as they feel they may cause permanent damage [9]. Instructions that can explain in simple terms that exploration on the app is welcome and can provide simple fixes to undo mistakes may help lessen this hesitancy.

Frustration stemming from the cognitive burden of difficult instructions or startup procedures, coupled with low trust in one’s ability to use mHealth, may also limit effective use [9,24]. Research staff often observed increases in frustration at certain points, such as unclear or shifting terminology or when they were not able to locate the correct button (eg, to activate Bluetooth). Research study staff noted that, for some individuals with low technology self-efficacy, these frustrations served to push them further away from acceptance and uptake of the device.

Of note within this study was that the barriers and frustrations experienced in many instances did not involve the technology app or device itself. Key points of friction included searching for directions, navigating changing terms, and struggling with customer support representatives to obtain answers they could understand. While much of the cognitive burden cited in the literature involves navigating the app itself [7], this precursor step of supporting materials is not often mentioned. However, this is a key point that overall user experience is vital when introducing an app [3]. Even small challenges such as a misplaced instruction sheet were enough to elicit negative emotions within several in this sample, which then accumulated over time as small setbacks increased at different points.

These impacts were concentrated mostly in those with lower initial technology skills and confidence. This observation might suggest that the impact of some irritations (eg, misplaced instructions) could be buffered to an extent by more confident users, whereas the same experience was interpreted as negative reinforcement of a lack of skills for others. In a past study, under technostress, high-stress participants experienced cognitive overload and, consequently, decreased performance, whereas low-stress participants felt moderate arousal and improved performance [25]. This may be reflected in the relatively steady intention to use the app beyond the study, where those with more positive perceptions and reactions to digital health indicated interest in further use, whereas those with higher stress and more negative reactions continued their refusal to future use.

This elicits a caution for future smartphone app developers that there may be differential weighting of issues perceived by those with varying levels of self-efficacy. A challenge that may be perceived as a minor inconvenience by some could be a key stumbling block for disengagement for others. Those impacts may change according to circumstances, with poor health reducing older adults’ ability to cope with technostress and/or increasing an individual’s struggles to deal with the constant evolution of digital technologies [26]. Future research should determine the stress-related factors and who might be most susceptible to their impacts, including those individuals who may feel that the cognitive load and treatment burden are too high for engaging with digital health solutions.

These experiences highlight the critical role that person-centered support and quality customer services can play in the app uptake and engagement process, particularly for those individuals who may have lower technological self-efficacy. Customer support interactions were often deemed unhelpful. The embarrassment felt by less technologically savvy participants compounded the issue as they felt too ashamed to say when they did not understand a direction. Future apps that have an older adult audience should provide more customer support training on clear instructions and proper terms, including not only what to do but also how to do it. Training customer support personnel on empathy and the impacts of aging would also contribute to improving the user experience of older adults. Stopping after each step to ensure that the user has successfully completed it may also put older participants at ease and help build confidence.

### Limitations

One limitation of this study was that many upstream barriers to technology engagement, including lack of internet access, cost, or awareness of the app, were removed for the participants to begin their journey with unboxing. Furthermore, the participant sample for this study had a self-identified behavioral health condition. Although this was an inclusion criterion for other potential study components, it does highlight the potential implications that those with a behavioral health condition may be even more susceptible to the impacts of technostress and anxiety. The sample comprised mostly White individuals, which is not representative of the larger older adult population. Future work should study these factors more in depth using a larger, more diverse sample of older adults studied over time.

### Conclusions

Digital health technology continues to advance and provide opportunities for older adults to engage with care and connected monitoring devices. Despite initial interest and excitement of the participants to unbox the tablet and explore the device, stress, frustration, and anxiety were present during the setup process, particularly for those with lower technology use. Key challenges of this process extended beyond the technology itself to include difficulties with terminology, paper instruction management, and ineffective customer support. Future studies should not ignore these crucial first impressions of technology as they may set the stage and prime emotions for future engagement and trust or lack thereof.

## Appendix

Multimedia Appendix 1 iCHECK-DH checklist.

## Abbreviations

BPM: blood pressure monitor
iCHECK-DH: Guidelines and Checklist for the Reporting on Digital Health Implementations
mHealth: mobile health
UTAUT: unified theory of acceptance and use of technology

## Data Availability

The datasets generated or analyzed during this study are not publicly available due to institutional policy but may be available from the corresponding author on reasonable request.
