# Supplementary material for: Older Adults’ Experiences Navigating Setup of Digital Health Technology: Implementation Report
Source: JMIR Form Res. 2026 May 4;10:e70319. doi: 10.2196/70319 (PMC13184594; doi:10.2196/70319)
Supplement: Multimedia Appendix 1 [file formative_v10i1e70319_app1.docx]

**Appendix 1.** Checklist of iCHECK-DH guidelines. iCHECK-DH: Guidelines and Checklist for the Reporting on Digital Health Implementations.

| section |  | Item | Description |
| --- | --- | --- | --- |
| Title | 1 | Title  (M^[[1]](#footnote-1)^) | We have reflected that the article is an implementation report in the title and keywords. |
| Abstract | 2 | Abstract  (M) | We have provided a summary of the implementation in the abstract, including a description of the implementation of the app. |
| Introduction | 3 | Context  (M) | Within our introduction section, we have identified barriers and facilitators to technology and app use by our target population (older adults in the US). We have identified our stage of implementation as piloting and evidence generation. |
|  | 4 | Problem statement  (M) | We have identified the gap in the literature regarding the detailed documentation  of the unboxing and first use of technology given the context described within the  introduction. We have also identified the classification of an “implementation forr persons” by the WHO. |
|  | 5 | Similar Interventions  (M) | While we are not directing adding to any specific study, our introduction cites similar findings in other work with older adults and technology. We highlight the need for more detail in the unboxing and first impressions of engagement with technology and apps. |
| Methods | 6 | Aims and Objectives  (M) | We describe the aims of this study (to explore in-depth reactions and barriers to initial engagement with a health app among a group of older adults). While specific KPIs were not tracked, we have structured our interview guide and approach based on the Unified Theory of the Use and Acceptance of Technology (UTAUT) to gauge effort expectancy, performance expectancy, and facilitating conditions. We also added health and technology questions based on initial formative research findings. |
|  | 7 | Blueprint summary  (M) | We have described the roadmap for study implementation, including the timeline for unboxing, onboarding, and qualitative assessments. |
|  | 8 | Technical Design  (M) | We have included a brief description of the tool used; however, we have not identified the selected tool as the focus was on overall technology perceptions and not the specific outcomes of that particular app. We have identified the app components (e.g., vitals monitoring and telehealth offerings) that were of priority to the organization. |
|  | 9 | Target  (M) | We have described our target population (older adults in Pennsylvania with mental health condition and low health management behavior) and the goals of observing their app use. |
|  | 10 | Data  (M) | We have described the data governance, including the storing, ownership, and protection of information, noting that the research team did not have access to the health data gathered through the app. We have included the consent process in the manuscript. |
|  | 11 | Interoperability  (M) | This was beyond the scope of the current study; relevant data operability standards will be evaluated by the organization once it is in the process of selecting specific apps to integrate into their offerings. |
|  | 12 | Participating entities  (M) | We have described the main participating entity (the healthcare organization) and acknowledged their marketing firm partner. We have also provided a funding statement to acknowledge the funder of this work. |
|  | 13 | Budget Planning  (M) | We have described the incentive structure for this study; however, budget for app integration with the organization’s health network (including any purchasing agreements, trainings, or system modifications) will depend on the actual offerings the organization selects to work within its system and is beyond the scope of the current article. |
|  | 14 | Sustainability  (M) | For the current study, we asked about the likelihood of continuing with the app if it were made available. This question, along with the other results on the perceptions of the app, will inform how the organization plans to approach sustainability during the selection and integration of future app offerings to its members. |
| RESULTS | 15 | Coverage  (M) | We have identified the current area as Southwestern Pennsylvania. |
|  | 16 | Outcomes  (M) | We have described the outcomes that have aligned with our framing of the UTAUT and other identified technology- and health-based factors. We have included an overall table that maps barriers and their potential solutions. |
|  |  | Lessons learned  (M) | Within our results, we included a table that had key barriers faced by participants and recommendations on how to address them in the future. In our discussion, we also discuss how the findings of this study could inform future app rollout for older adults. We also discuss the limitations of the current study, and the value of extending this work to a more diverse population of older adults. |
|  | 17 |  |  |
|  | 18 | Unintended consequences  (NM^[[2]](#footnote-2)^) | There were no unintended consequences of this study. |
| Discussion | 19 | Conclusion  (M) | We have included a conclusion paragraph and have discussed future implications of this work to consider the very first stages of app introduction in future technology-based interventions with older adults. |
| General | 20 | General  (NM) | We have included an ethics section and have identified the presiding IRB board for the study. |

1. M: Mandatory item [↑](#footnote-ref-1)
2. NM : Non-mandatory item [↑](#footnote-ref-2)
